# In Situ Fabrication of TiC/Ti–Matrix Composites by Laser Directed Energy Deposition

**DOI:** 10.3390/ma17174284

**Published:** 2024-08-29

**Authors:** Sabin Mihai, Florin Baciu, Robert Radu, Diana Chioibasu, Andrei C. Popescu

**Affiliations:** 1 Center for Advanced Laser Technologies (CETAL), National Institute for Laser, Plasma and Radiation Physics (INFLPR), 077125 Magurele, Romania; sabin.mihai@inflpr.ro; 2 Faculty of Industrial Engineering and Robotics, National University of Science and Technology Politehnica Bucharest, 060042 Bucharest, Romania; florin.baciu@upb.ro; 3 Faculty of Applied Sciences, National University of Science and Technology Politehnica Bucharest, 060042 Bucharest, Romania; radurobert@yahoo.com

**Keywords:** titanium matrix composite, titanium carbide, direct energy deposition, laser beam, robotic arm

## Abstract

In this study, crack-free TiC/Ti composites with TiC content ranging from 0 to 15 wt.% were successfully fabricated using Direct Energy Deposition with a dual-feeder system that concomitantly delivered different amounts of both constituents into a high-power laser beam. The samples were investigated to evaluate the morphologies and distribution behavior of TiC. The microhardness values of the samples obtained under optimal processing conditions increased from 192 ± 5.3 HV_0.2_ (pure Ti) to 300 ± 14.2 HV_0.2_ (Ti + wt.% 15 TiC). Also, TiC has a significant impact on the Ti matrix, increasing the strength of TMCs up to 725 ± 5.4 MPa, while the elongation drastically decreased to 0.62 ± 0.04%. The wear rate is not proportionally affected by the rise content of TiC reinforcement; the hypoeutectic region of TMCs exhibited a wear rate of 2.45 mm^3^/N·m (Ti + wt.% 3 TiC) and a friction coefficient of 0.48 compared to the ones from the hypereutectic region, which measured a wear rate of 3.02 mm^3^/N·m (Ti + wt.% 15 TiC) and a friction coefficient of 0.63. The improved values of mechanical properties in the case of TMCs as compared to pure Ti are provided due to the solid solution strengthening of carbon and the fine grain strengthening. This work outlines a method for changing TiC morphologies to improve the hardness and tensile strength of TMCs fabricated starting from micro-scale powder.

## 1. Introduction

Metal Matrix Composites (MMCs) are materials that consist of a metallic matrix reinforced with a dispersed phase of metallic, ceramic, or polymer in the form of micro- or nanoparticles, tubes, or whiskers [1], which surpasses the physical and/or mechanical properties of the matrix metal. Thus, materials with light weight and excellent mechanical properties can be obtained, with great potential to revolutionize engineering fields such as aircraft, defense, aerospace, or automobiles [2]. The most common traditional methods of obtaining these MMC materials are powder metallurgy and in situ casting [3]. These techniques present specific problems that cannot be avoided, such as the low wetting between the ceramic materials and the metal matrix, which leads to the deterioration of the ductility of MMCs or the segregation of ceramic particles in the matrix during the obtaining process. The microstructure of such materials is characterized by very small grains, which represents an exponential increase in grain boundaries as compared to casted materials [4]. In these cases, the heat concentrates at grain boundaries, which makes the machining and welding of bulk MMC extremely difficult [5,6]. There are solutions for machining and welding of MMC, but they are expensive and consequently destined for luxurious transport vehicles. The necessity of identifying a technique to cheapen the manufacturing costs of parts made of MMCs for the benefit of industries devoted to mass consumption is the main objective of this research. The perspectives of implementation of such materials in numerous fields of industry are unlimited: cars with light bodies for low fuel consumption and high driver protection, bicycle lighter frames, friction parts that last longer, such as gears or fins, buildings with more elastic and resistant frames, implants, and prostheses with prolonged lifetimes.

Titanium and its alloys are the most common materials used in aerospace due to their physical-chemical properties: excellent high-temperature stability, high specific strength, and good corrosion resistance [7,8,9,10,11]. However, with the continuous development of society and science, materials need to respond to stringent service conditions and harsher requirements. By adding a dispersed phase of particles or fibre materials in the Ti bulk, one can drastically alter some of its properties. Titanium Carbide (TiC) possesses high hardness, good thermal stability, and good compatibility with titanium and its alloys, mostly due to its similar density to titanium. MMC reinforced by TiC particles usually possesses superior strength, hardness, and fatigue resistance compared to the titanium properties [12,13,14,15,16,17]. This particular type of material demonstrates potential for application in the fabrication of turbine blades and aerospace turbine components (thrusters and spray nozzles), owing to its commendable physico-mechanical properties [18,19]. The material possesses noteworthy potential for application in the defense sector, specifically in the production of lightweight, hard plates for body armor systems. Furthermore, these MMCs exhibit interest in the manufacturing of prosthetic joints as well due to the favorable characteristics of the Ti matrix, encompassing high bio-compatibility, in conjunction with the wear-resistant properties of TiC [12]. Robotic arms have also garnered significant interest due to the enhanced reach and precision in operation achieved through the utilization of MMCs, characterized by their high stiffness and lighter weight [20].

Direct energy deposition (DED) [21,22] has proven to be a flexible technique regarding the additive manufacturing process for the production of MMC materials without internal defects such as pores, cracks, or lack of adhesion between materials, since the process allows for simultaneously feeding different powders with various compositions into the molten pool generated by the laser beam. The layer-by-layer manufacturing strategy, specific to the DED method, helps obtain dense materials with uniform homogenization. In addition, using this manufacturing method, components with complex geometry and dimensions very close to those written in the technical drawing can be made. By using the DED technique, parts with outstanding mechanical properties and complex geometries can be obtained in one step of fabrication. The process has been successfully used to generate bulk components to near-net shape tolerances with improved hardness [23], strength [24], wear, and corrosion resistance [25] in comparison with conventional fabrication methods. Functionally graded materials (FGMMC) can also be produced by adjusting the powder feed rates during the deposition process [26].

Numerous studies on various types of MMC manufactured by DED have been recently published by researchers all over the world [27,28,29]. TiC particle content dispersed in the Ti matrix is the main parameter that directly affects the microstructure and properties of the metallic matrix. Wang J. et al. [14] investigated the effect of TiC particle size on the microstructure and tensile properties of TiC/Ti6Al4V composites fabricated by DED. They demonstrated that the large size of undissolved TiC particles and their defects reduce the strength of composites by affecting the microstructure of the deposited material. Yu C. et al. [30] studied the impact of using high laser power during processing and discovered that this can facilitate the dissolution of TiC. Liu S. et al. [31] investigated the influence of the TiC reinforcement particle melting degree on the microstructure and mechanical properties during laser-directed deposition of Ti6Al4V-TiC composites. It was reported that the melting degree of TiC particles (TiCp) could be adjusted by controlling the laser energy density. One of the main difficulties of producing in situ MMCs is the weak bonding between the reinforcement particles and the metallic matrix due to the high melting point of the ceramic particles. Schopphoven T. et al. [32] proposed a particular high-speed DED approach by increasing the interaction distance between the laser beam and powder debit, which accelerated the powders melting. Sun X. et al. [33] produced in-situ TiC-reinforced titanium matrix composites by hot-press sintering and hot rolling. They studied the interfaces between in-situ reinforcements and the matrix in order to characterize the bonding level. It has been found that the size of ceramic particles influenced the microstructure and properties of MMCs. Reinforcing the Ti matrix with TiC ceramic particles improves the mechanical properties of the structures, such as rigidity, hardness, and wear resistance, while at the same time keeping its density low [34,35]. In the literature, this type of MMC is known as Titanium Metal Matrix Composites—TMC [34]. It has been demonstrated that a process with a high solidification speed is suitable for manufacturing these types of composite materials with Ti matrix [27], which makes the DED technique suitable for this kind of application due to the non-contact fast heating-cooling process [36].

The objectives of the current study are: i. synthesize TMCs with a dispersed phase of TiC by DED; ii. investigate the effect of TiC content on the phase evolution of the titanium; and iii. optimize the TiC content and provide essential information for further composition tuning. The microstructure, phase evolution, hardness, tensile strength, and wear resistance of TiC dispersed titanium TMCs will be analyzed and discussed. Moreover, the strengthening mechanism of the composite will be further analyzed and discussed.

## 2. Materials and Methods

### 2.1. Experimental Set Up 

Metallic material used for this study was Spherical Ti powder (wt.% 99.85 Ti) and ceramic polyhedral TiC powder (wt.% 79.95 Ti, wt.% 20.05 C), with particle dimensions between 45–150 µm and 45–106 µm, respectively. Both types of powder were acquired from Atlantic Equipment Engineers, Inc. (Upper Saddle River, NJ, USA). The substrate used for experiments was a commercially pure Ti plate of 10 mm thickness.

**Figure 1:**
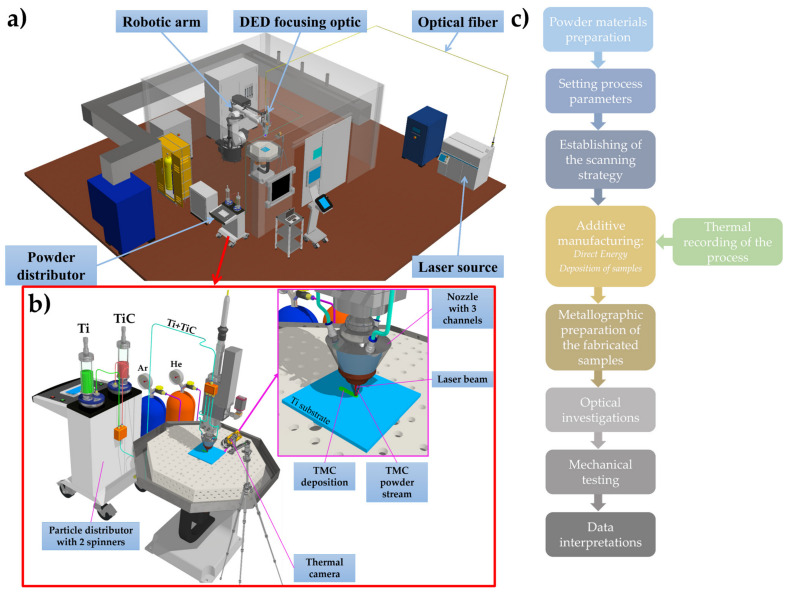
(**a**) Schematic of the experimental set-up used for manufacturing composite materials with Ti matrix and TiC dispersed phase in form of particles; (**b**) schematic of the powder distribution using two spinners; and (**c**) flowchart of the experimental stages.

The two types of powders were introduced separately into a particle distributor with 2 spinners (GTV, Verschleißschutz GmbH, Luckenbach, Germany), which delivers the materials through hoses (Ø = 6 mm) to a focusing optics mounted on a robotic arm (KR30HA, Kuka, Augsburg, Bavaria, Germany). One cylinder of the feeder was filled with Ti sphere-shaped powder, while the second cylinder contained solely TiC powder (Figure 1). The powder was transported by He used as carrier gas (3 slpm) mixed with Ar as protective gas (10 slpm). The gas mixture had the role to ensure a uniform distribution of the powder flow and a proper gas shroud around the working area that protected the deposited sample against oxidation. The mixed deposited material was produced in situ by calibration of the powder debit. The laser beam melted the powders and formed the composite material in liquid phase, which solidified rapidly after the stop of the laser action. An Yb:YAG thin disk laser source (λ = 1030 nm) with continuous emission, a “top-hat” energy distribution, and a diameter of the focused laser spot of 800 µm, using a power of 700 W (optimized in a previous study for melting the powders and producing bulks without structural defects such as pores or cracks [37]), was able to melt the TMC in order to obtain defect-free depositions. By simultaneously translating the laser beam and the powder jets, a wall of deposited material could be grown on the substrate. The optimal translation speed was found to be 10 mm/s, to allow for tracing of clearly defined lines with parallel borders and uniform distribution of deposited material. By repeating the same contour and controlling the laser beam focus, the deposition resulted in a 3D object, built layer by layer in this manner. The hatch spacing was set at 1 mm and offset between layers at 0.5 mm.

The melt pool generated during the DED process was monitored using a thermographic camera TIM M-05 (Micro-Epsilon, Ortenburg, Germany) with sensitivity in the range of 900–2450 °C. The thermal camera works in the spectral range λ = 500–540 nm, at a frequency of 80 Hz and resolution of 382 × 288 pixels. The value of a pixel was determined based on the distance from the camera to the zone of interest and can be calculated using a program provided on the manufacturer’s web page optics calculator for thermoIMAGER TIM [38]. During the experiments, the thermal camera was placed at a distance of ~975 mm and on a slope of 55°, which covers a ~25 × 15 cm^2^ area of monitoring. The dimension of a pixel was 580 µm.

For this study, TMC’s deposition was obtained with wt.% 1, wt.% 2, wt.% 3, wt.% 5, wt.% 10, and wt.% 15 TiC concentrations. Before the DED process, both powders were submitted to a thermal treatment at 60 °C for 4 h in an oven to eliminate the adsorbed water and then were introduced directly into the two cylinders of the particle distributor. The first cylinder contains pure Ti powder, which serves as the metal matrix, while the second cylinder contains TiC powder, acting as the dispersed phase in the form of particles with a reinforcing role. To determine the desired TiC concentrations, we calibrated the powder delivery rates for both Ti and TiC. For example, for one rotation per minute (rpm) of titanium, the delivery rate is 1.92 g/min, while for TiC, it is 0.69 g/min. Both types of materials in powder form were simultaneously delivered through hoses and directed into the laser beam via a nozzle with predetermined flow rates, which were established according to the rotations per minute of each powder material.

### 2.2. Microstructure Characterization

The Ti and TiC particle morphology was investigated by Scanning Electron Microscopy (SEM) using a Quanta Inspect S (Eindhoven, The Netherlands) system. The physical properties of the base material and powders are presented in Table 1.

After performing the deposition experiments, all the samples were subjected to metallographic and optical microscopy characterizations. For this purpose, a Brilliant 200 cutting machine (ATM, Mammelzen, Germany) with a disc rotation speed of 2850 rpm was used. The sliced specimens were then embedded in bakelite using a hot-pressing machine with a 30 mm diameter, 50 bar pressure, and 200 °C temperature, employing an Opal 410 device (ATM, Mammelzen, Germany). Finally, the specimens were polished to mirror-like level using a Saphir 520 machine (ATM, Mammelzen, Germany). The samples were etched with Kroll reagent for 45 s in order to reveal the grains and the constituent metallographic phases. The microstructure of the deposited materials was analyzed by optical microscopy using a Leica DM2700M RL (Wetzlar, Germany) with LED illumination, equipped with four different microscope objectives that had a magnification power of 5×, 10×, 20×, and 50× coupled to a Leica MC190 HD digital camera, and by SEM using a Quanta Inspect S system (Eindhoven, The Netherlands). The areas of the irregular shapes of granular eutectic, primary phase, and unmelted TiC particles were quantitatively determined by image analysis of optical microscopy and SEM images and by using open-source image processing software (ImageJ 1.54i 03—LOCI, University of Wisconsin, Madison, WI, USA). Three images were used for each sample. The values presented for TiC microstructure are the average values of the three images for each sample.

**Figure 2:**
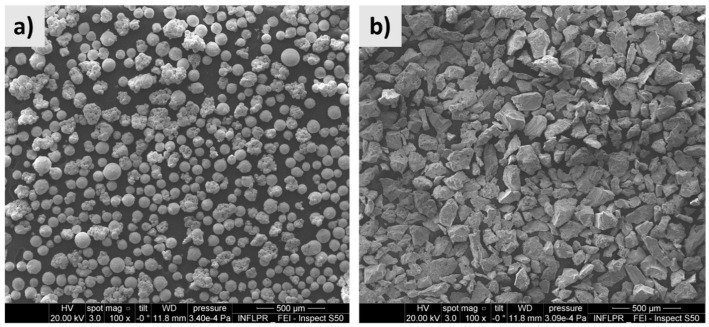
SEM imaging at the magnification of 100× of Ti (**a**) and TiC (**b**) powder particles.

Figure 2a presents a typical SEM micrograph at the magnification of 100 of the spherical Ti powder and some parasite particles with diameters between 5–10 µm that coalesced and formed irregular-shaped grains with sizes up to 150 µm. The TiC powder (Figure 2b) was polyhedral, with irregular shapes and dimensions that reach up to 106 µm, allowing for a constant flow of material through hoses.

### 2.3. Mechanical Properties

Microhardness was analyzed by using the scratch test method, and tests were conducted using an automated multi-function tribometer (Rtec–Instruments, San Jose, CA, USA). All experiments were conducted in air at ambient conditions with temperature and relative humidity (RH) maintained at 22.7 °C, respectively 40% RH, using a Rockwell diamond stylus with a tip radius of 200 μm. The test was performed in accordance with ASTM G171–03. Three different normal loads of 25, 50, and 75 N were applied during scratching at a constant sliding speed of 0.15 mm/s. The length of each scratch segment was set at 3 mm, and the samples were indented under constant load with the specific indicated normal force. After tests, the scratched surfaces were analyzed using a post-scanning operation with an in-line 3D confocal microscope (Rtec–Instruments, San Jose, CA, USA). The hardness value was calculated by dividing the applied normal force on the hemi-spherical stylus with a radius curvature “*r*” by the projected area of scratching contact. The projected area of the contact surface had the same diameter as the final scratch width. Thus, using Equation (1), the hardness of each deposited TMC can be determined:

(1) $${HS}_{p}=\frac{8P}{{\pi \cdot w}^{2}}$$

where: HS_p_ = scratch hardness (Pa), P = normal force (N), w = scratch width (m).

The friction and wear resistance properties of the deposited pure titanium and TMC composites fabricated by DED were tested. The dry sliding tests were conducted on a rotating ball-on-disk tribometer (MFT, Rtec–Instruments, San Jose, CA, USA) with an adjustable trajectory radius. Prior to the tribology tests, all specimens were mirror-like polished in order to control the average surface roughness (Ra). The metallic ball Steel 440C with 6 mm diameter was used as the counterpart. Wear tests were performed at 60 N with constant sliding speed maintained at 150 rpm on a 10 mm circular trajectory for 5 min. All the wear tests were conducted in air at room temperature of 22.7 °C and the ambient dry condition of 40% RH. During the tribology test, the instantaneous shear force (Ft) was recorded and monitored using the software of the equipment, which is also able to trace the curve of the friction coefficient in real-time. Topographical profiles of the wear track were measured for each TMC sample. The mass loss (M_L_) is given by M_L_ = wear track cross-section area × track length, where the wear track cross-section area is calculated by integrating the average wear track profile. The morphology of the worn surface was characterized by 3D confocal microscopy.

The 3D specimens with specific shapes for tensile tests were printed. The tests were performed at room temperature using an Instron 8872 machine (Norwood, MA, USA). The specimens were fixed between the two jaws of the machine, and the upper jaw was triggered with an increasing longitudinal force until failure.

To ensure the reproducibility of the measurements, both mechanical analyses were conducted in triplicate. Mechanical—physical properties of Ti and TiC materials are presented in Table 2.

### 2.4. Scanning Strategy

The first stage in the optimization of the geometric parameters of the scanning strategy was the printing of parallel pipes of 30 × 50 mm. We identified the optimal distance between hatch lines and layers that leads to obtaining straight edges and a value within tolerance for the experimental height. This step is essential to ensure the 3D parts have geometrical dimensions close to those inscribed in the technical drawings. These critical parameters were 1 mm for hatch distance, 0.5 mm between layers, and kept constants for all 6 TMC material compositions.

## 3. Results and Discussion 

### 3.1. TMCs Microstructure

**Figure 3:**
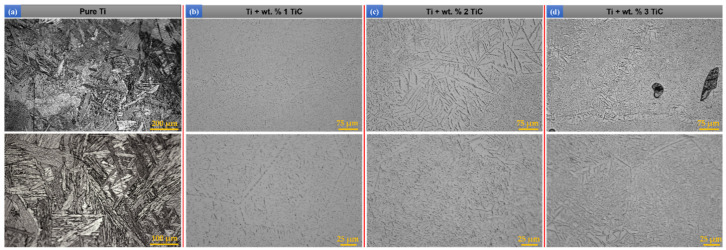
Optical microscopy images of DED-deposited bulks of (**a**) Ti, (**b**) Ti + wt.% 1 TiC, (**c**) Ti + wt.% 2 TiC, and (**d**) Ti + wt.% 3 TiC.

In Figure 3, the microstructures of the bulk deposited samples by DED of Ti and TMC with wt% 1, wt% 2, and wt% 3 TiC content are presented. The pure Ti-deposited structure, used as a control, was identified as α + β type, with interwoven acicular grains of hexagonal compact α phase grown in large polyhedral grains of β phase (cubic with centered volume). The α grain superposition formed a basket-shaped Widmansttaten structure (Figure 3a), inserted in the β-phase large grains. Due to repeated irradiation of the same area by laser processing, the temperature rose above 890 °C, which represents the threshold α > β transformation, and therefore the dark grains of the β phase are identified between the light-colored α grains. In the case of TMC, the α-Ti phase grains were interwoven inside the β-Ti grains, which results in a microstructure with a higher proportion of β-phase. There are areas where acicular α-Ti grains nucleated over other grain colonies, contributing to the formation of the Widmanstatten structure.

The TiC particle concentrations in the range of wt.% 1–3 in the Ti metal matrix caused a minimal impact on the phase transformation of typical pure Ti microstructure. According to the Ti-C binary phase diagram, the carbon content of the eutectic point is about 2%. The composites with wt.% 1–3 TiC in bulk (Figure 3) are in the range of hypoeutectic area because the TiC volume fraction is below 5% according to the Ti-C phase diagram. Due to the fast process of cooling down the melt pool from high temperature (~1400 °C, which is very close to the melting point of the pure Ti) to a value under 400 °C, the β-Ti phase will be solidified firstly from the liquid state of pure titanium because of the differences between the solid states of each phase (the melting point of the TiC is ~3140 °C). In the solidification process of the β-Ti phase, the carbon concentration decreases to the eutectic point, and the TiC + β-Ti eutectic reaction occurs. When the temperature drops to β-transus temperature (~890 °C), the β-Ti transforms to α-Ti. However, the rapid cooling rate characteristic in the DED process during solidification makes impossible the β-Ti complete transformation into α-Ti.

**Figure 4:**
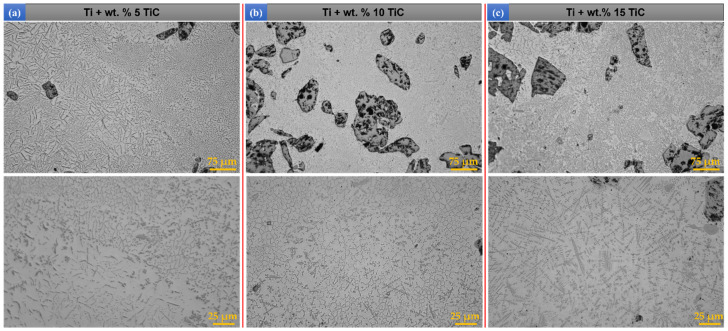
Optical microscopy images of DED-deposited bulks of (**a**) Ti + wt.% 5 TiC, (**b**) Ti + wt.% 10 TiC, and (**c**) Ti + wt.% 15 TiC.

Figure 4 presents the TMCs that were manufactured by DED with a larger amount of TiC particles. Figure 4a displays the TMC microstructure with a content of TiC up to wt.% 5, and it can be observed that the primary dendritic TiC occur. The increase in Carbon due to the step-up to wt.% 10 TiC provides the necessary mass for an increase in the TiC dendritic dimensions and the number of unmelted particles that can lead to crack formation (Figure 4b). This phenomenon is further enhanced in the bulk sample with wt.% 15 (Figure 4c), where the primary dendritic phase becomes significantly coarser and it can be observed the apparition of secondary and tertiary dendrites.

In case of changing the morphology of the matrix, there are two possible reasons: i. the pure titanium powder may contain few impurity elements and can influence the growth rate of the grains after the nucleation stage during the cooling process, and ii. the addition of TiC particles affects the cooling rate of the melt pool due to the high laser energy absorption rate. Therefore, the difference in the microstructure of the deposited material obtained in the experiments presented in this study indicates that the cooling rate of the molten pool of samples with lower concentrations of TiC may be faster than that of the TMC molten pool with higher concentrations of TiC particles.

Because the processing parameters (laser power, scanning speed, focusing distance, gas mixture flow) were kept constant, the cooling rate differences could be assigned to the reflectivity coefficient for the laser beam of TMC composites with different TiC particle content. The laser absorption coefficient of a material depends in general on its material resistivity [39]. Thus, when the laser wavelength is constant, the absorption rate increases with the increase of the material resistivity. In this case, the material resistivity of TiC (105 μΩm) is significantly higher in comparison with the one of commercially pure Ti (0.554 μΩm). This indicates that the TiC powder has a higher laser absorption coefficient; hence, by increasing the concentration of TiC in the Ti matrix, the laser absorption coefficient of the powder mixtures will increase and the temperature of the molten pool will be higher. The reaction for the synthesis of TiC is exothermic, and a large amount of heat is released during the deposition of TMCs.

Examination of the sample’s microstructures by SEM at higher magnification revealed numerous TiC phases, depending on the TiC particle content, that are spread in the entire volume of the TMCs.

Due to the layer-by-layer manner-specific technique for 3D printing using the DED method, after each layer, the surface of the last deposited layer will partially be remelted, and some compositional differences between the interface area and the bulk can emerge. For TMCs, two mechanisms of integrating the TiC particles in the metal matrix were identified: (i) TiC will partially melt and re-solidify as dendrites, which refines the matrix microstructure, and (ii) the TiC particles will remain unmelted within the bulk due to the insufficient laser energy delivered in the melt pool. In the case of DED, it is difficult to distinguish the interface between layers and the remelted area within each layer. The high absorption of the laser beam, which led to a higher temperature of the molten pool, caused the dissolution of the TiC particles. If the TiC content increases in the bulk, the mean particle dimensions and the quantity in the bulk will also increase, leading to the apparition of a microstructure that includes fine unmelted or remelted particles in the metal matrix volume.

**Figure 5:**
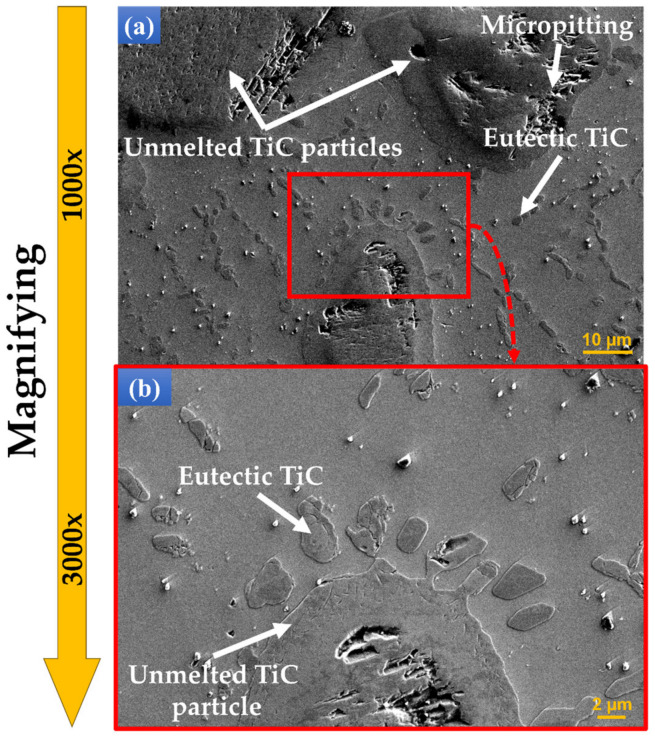
SEM investigation of the TiC microstructure: (**a**) at the 1000× magnification and (**b**) at 3000× magnification.

Figure 5 presents a strong adhesion without the presence of any defects at the interfaces between the TiC microstructure and Ti matrix. In addition to the melting of TiC particles, the partial dissolution of TiC particles into the molten pool during the DED process could also be obtained. The level of TiC dissolution was influenced by the liquid metal matrix and TiC interaction time with the laser energy, which is low due to the fast processing cycles. In consequence, it was difficult to melt/dissolve into the Ti matrix the entire TiC particles in TMCs with a content above wt.% 3 TiC. Therefore, most of the samples with an increased percentage of TiC were partly melted, and some unmelted TiC particles were trapped in the Ti matrix, as described in Figure 4.

**Figure 6:**
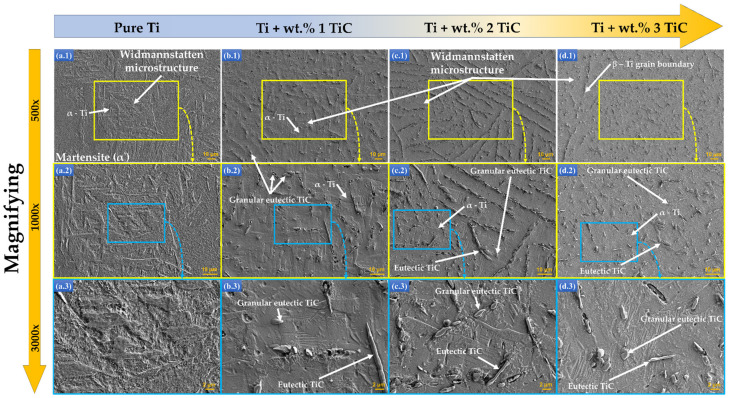
The metallographic morphologies of the DED-deposited TMCs in the component of (**a.1**–**a.3**) commercially pure Ti, (**b.1**–**b.3**) wt.% 1 TiC, (**c.1**–**c.3**) wt.% 2 TiC, and (**d.1**–**d.3**) wt.% 3 TiC investigated by SEM under different magnifications of 500× (**a.1**–**d.1**), 1000× (**a.2**–**d.2**), and 3000× (**a.3**–**d.3**).

For a better understanding of the TiC microstructure, Figure 6 presents the SEM images at different magnifications starting from 500 (Figure 6(a.1–d.1)), continuing with 1000× (Figure 6(a.2–d.2)), and up to 3000× (Figure 6(a.3–d.3)) of the microstructure of TiC/Ti composites with percentages of TiC particles between wt.% 1–3. As exhibited in Figure 6, the microstructure of metal matrix in TMCs with different content of TiC particles appeared to be Widmannstatten microstructure. It could be seen from Figure 6(b.1–d.3) that the reinforcements of TiC/Ti composites were the granular and chain-shaped eutectic phases, granular primary TiC, and a reduced number of unmelted particles. The phases were homogeneously spread in the metal matrix.

The concentration increase from wt.% 5 to wt.% 10 TiC caused the appearance of a main primary dendritic structure, a secondary dendritic structure that evolves from the main one, and a granular component, which is more significant for 10 than for wt.% 5 TiC in the Ti matrix (Figure 4b). In the case of wt.% 15 TiC (Figure 4c), the secondary dendritic phase is overdeveloped, and also the highest number of undissolved TiC particles in the Ti matrix were identified.

**Figure 7:**
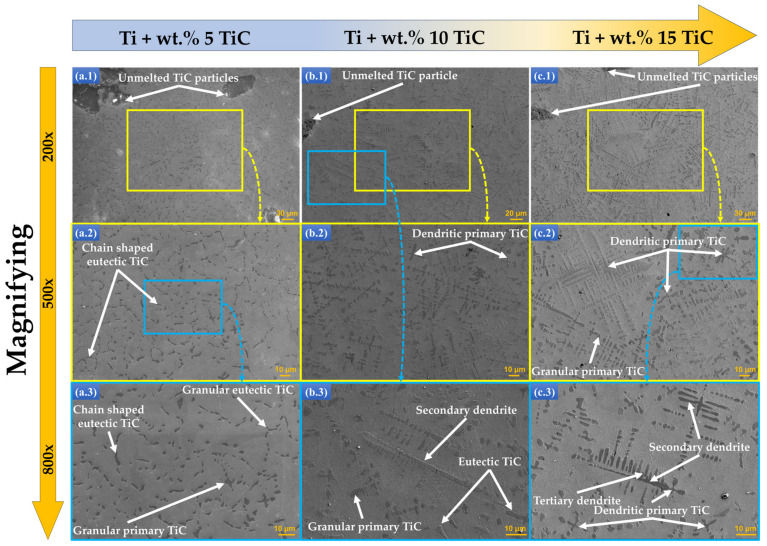
The metallographic morphologies of the DED-deposited TMCs in the components of (**a.1**–**a.3**) wt.% 5 TiC, (**b.1**–**b.3**) wt.% 10 TiC, and (**c.1**–**c.3**) wt.% 15 TiC investigated by SEM under different magnifications of 200× (**a.1**–**c.1**), 500× (**a.2**–**c.2**), and 800× (**a.3**–**c.3**).

The metal powder particles of commercially pure Ti would be initially melted during the laser processing. This is attributed to the fact that the melting point of Ti matrix (~1668 °C) is significantly lower than that of TiC used as reinforcement phase of materials (~3140 °C). The TiC reinforcement particles would be completely melted if the temperature value of the molten pool was closer to one of the TiC melting points. The microstructure of deposited samples presented in Figure 6 reveals that the size of unmelted TiC particles from the TMCs that are in the hypoeutectic region is in the range of 0.23 mm^2^ to 5.25 mm^2^. In the case of the samples exhibiting in Figure 7 TMCs from the hypereutectic region, the unmelted particles are in the range of 0.27 ± 10.95 mm^2^.

By varying the TiC content in the TMC materials, it leads to different solidification conditions of the molten pool. Different types of TiC phases are formed during the solidification process. Thus, three main forms of TiC morphologies appear: unmelted particles, eutectic, and primary phases. It is observed from Figure 3 and Figure 4 that the morphology and size of the precipitated TiC phases at different TiC content are not consistent. When the TiC mass fraction is relatively low, under wt.% 5, chain-shaped eutectic TiC and granular eutectic TiC phases can be identified (Figure 7(a.1–a.3)). In addition, it can be observed that the chain-shaped TiC phase has a tendency to line up at the grain boundaries of β-Ti. As the content of TiC gradually increases, the chain-shaped and granular eutectic TiC phases gradually decrease, while the granular and dendritic primary TiC phases gradually increase. It can be observed from Figure 7(b.1–b.3) that the dendritic primary TiC phase at wt.% 10 TiC is insufficiently grown. As the TiC content continues to increase up to wt.% 15, the size of the granular primary TiC phase keeps increasing, and the dendrites become coarser, showing secondary and tertiary dendritic arms (Figure 7(c.1–c.3)).

**Figure 8:**
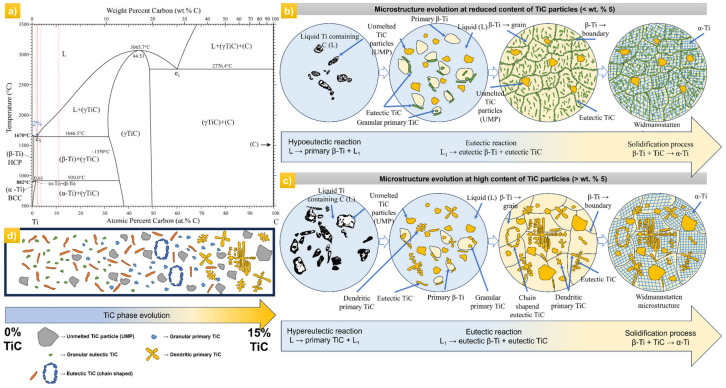
Schematic diagram of the microstructure evolution during the LMD process: (**a**) phases and mixtures in the Ti-C system; (**b**) the TiC morphologies evolution in the hypoeutectic or eutectic region at low content of TiC (below wt.% 5 TiC); (**c**) the TiC morphologies evolution in the hyper-eutectic region at high content of TiC (over wt.% 5 TiC); and (**d**) microstructure variation of the TMCs depending on the TiC content percentage.

In order to discuss the mechanism of microstructure formation in depth of titanium matrix composites with different content of TiC reinforcement particles, the schematic representation of phase formation is presented in Figure 8. The disk laser used for the manufacturing of the composite materials had a circular cross section with a “Top-Hat” intensity distribution, which was expected to generate a quasi-uniform high temperature gradient in the molten pool.

As presented in Figure 8, the schematic diagram exhibits the microstructure evolution below and above wt.% 5 TiC. When the TiC content in TiC/Ti depositions is low, the TMC composition is in the hypoeutectic or eutectic composition region, according to the titanium-carbon (Ti-C) phase diagram [40] as shown in Figure 8a. In this case, during the cooling process, the metallographic phases of the materials are sequentially primary β-Ti + liquid phase that include the nucleation of another two phases (chain-shaped eutectic TiC + granular eutectic TiC) and α-Ti phase zones in the interior of β-Ti grain as the temperature decreases. The chain-shaped and granular eutectic TiC have a tendency to align at the boundary of the β-Ti grain. When the heat of the deposition reaches 1646 °C, eutectic reactions appear in the molten pool and generate eutectic TiC and β-Ti. After the laser processing ends, the temperature of the melt pool progressively decreases, and β-Ti precipitates during the cooling process. Once the temperature drops up to β-Ti transition point (~890 °C), solid-state phase transition takes place within each β-Ti grain, finally forming a α + β two-phase structure in the matrix. Because of the high cooling rate characteristic of the DED process, α-Ti precipitates through non-equilibrium martensitic transformation, thus leading to the Widmannstatten structure [41]. The schematic diagram of the microstructure evolution at a reduced content of TiC is presented in Figure 8b. In this case, in-situ eutectic TiC phase precipitates with the microstructure of short tubes, granular and chain-shaped. These types of morphology were also identified by Zhang J. et al. at different experimental conditions and TiC content [42].

Figure 8c illustrates the microstructure evolution of the TiC phase in the TMC composite as the TiC content is over wt.% 5. When the TiC content in Ti/TiC depositions is higher, the TMC composition is in the hypereutectic composition region, according to the titanium-carbon (Ti-C) phase diagram. Correspondingly with the first case, the liquid melt pool that contains carbon is generated with the melting of Ti powder and the dissolution of TiC particles. During the cooling process, with the carbon concentration in the melt pool increasing through diffusion, the hypereutectic reaction between Ti and C materializes, and then primary TiC phases are precipitated from the liquid Ti matrix, as is indicated in eq. L → primary TiC + L1. It develops into grains with different shapes and sizes depending on the TiC content. The primary TiC phase does not solidify at the same time, thus newly formed granular, chain-shaped, and dendritic shapes occur. The secondary phase of dendritic TiC is commonly supposed to be caused by the thermal variations at the dendrite surface. The tertiary TiC dendrites are mainly oriented by the thermal gradients in the melt pool. While the temperature decreases, the β-Ti grains keep growing until they occupy the whole matrix, the primary TiC stops precipitating, and the eutectic reaction occurs as in eq. L1 → eutectic β-Ti + eutectic TiC, followed at the end by the β-Ti to α-Ti transformation [41]. The α-Ti dendrites are distributed inside the β-Ti grains, developing a Widmannstatten microstructure. In this case, the enhanced carbon activity and the extended reaction time assure the development conditions of dendritic primary TiC phases. Thus, due to the high content of C in the melt pool, more secondary and even tertiary dendritic arms are formed. The specific microstructure of this type of material was also validated by L. Li et al., but at a content higher than wt.% 20 TiC [19].

According to the analyses of the metallographic morphologies of TMCs with different TiC content, the TiC powders could bear considerable melting and dissolution during the DED processing (Figure 6 and Figure 7). The increase in TiC fractions provides more C that allows the formation of primary TiC. Based on the Ti–C phase transformation diagram, a rise in the carbon content results in a pronounced elevation of the liquidus line and an expanded temperature range for primary TiC precipitation. Thus, a higher degree of undercooling at the front interface of primary TiC will be expected. This phenomenon promotes the formation of primary TiC phases with dendritic shapes. Even slight turbulences from heat gradients at the solid-liquid interface during the growth process of TiC give the possibility of secondary and tertiary dendrite growth. In the case of the eutectic and primary TiC phases, both apparitions are referred to as resolidified TiC. In order to clearly understand the microstructure of the TMCs depending on the TiC content, the diagram of each composition up to wt.% 15 TiC is illustrated in Figure 8d.

### 3.2. Particle Distribution Analysis

**Figure 9:**
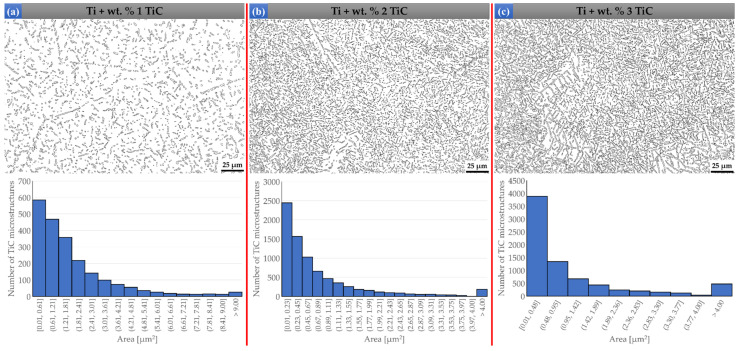
TiC microstructure images of TMC samples and area values of TiC phase distribution for concentrations with wt.% 1 TiC (**a**), wt.% 2 TiC (**b**), and wt.% 3 TiC (**c**).

**Figure 10:**
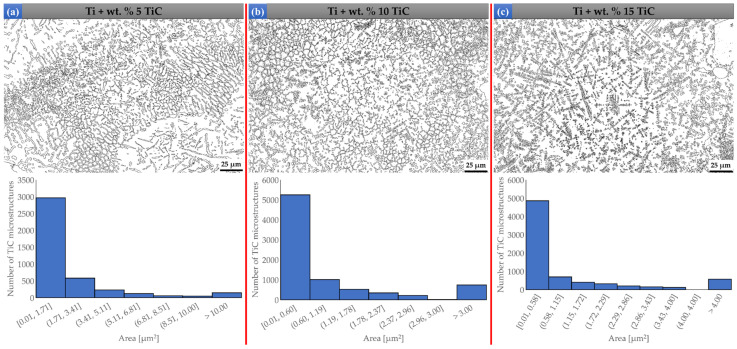
TiC microstructure images of TMC samples and area values of TiC phase distribution for concentrations with wt.% 5 TiC (**a**), wt.% 10 TiC (**b**), and wt.% 15 TiC (**c**).

The microstructure of pure Ti deposition is composed of α-Ti + β-Ti Widmanstatten structure characterized by α needle grains that grow from β grains in multiple directions and intertwining. This type of microstructure corresponds to the purity of the used powder (>99.95%). The results obtained for the sample with wt.% 1, 2, and 3 TiC are shown in Figure 9. For the TMC with a concentration of wt.% 1 reinforcement material, the TiC microstructures have an average area of 1.87 µm^2^ (Figure 9a). The increased content of Carbon due to the raised percentage of TiC up to wt.% 2 exhibits a similar type of microstructure, and the mean value of the particle area decreased to 0.81 µm^2^ (Figure 9b). Therefore, the reinforcing structure became significantly well spread in the Ti matrix. This phenomenon is further magnified in the bulk sample with wt.% 3, where the mean area of the TiC phase is equal to 1.23 µm^2^ (Figure 9c). When the content of TiC goes beyond wt.% 3, the number of unmelted particles significantly increases. The results pertaining to samples containing wt.% 5, wt.% 10, and wt.% 15 TiC are also outlined in Figure 10. Notably, the sample with wt.% 5% reveals a TiC microstructure with an average area of 2.23 µm^2^ (Figure 10a). When the content of TiC is increased up to wt.% 10, the microstructure shows an intensification in primary dendritic TiC, and the mean particle area is reduced to 1.15 µm^2^ (Figure 10b). Subsequently, the bulk sample containing wt.% 15 TiC demonstrates a mean TiC phase area of 1.2 µm^2^ (Figure 10c). Notably, beyond wt.% 10% TiC, the number of unmelted particles can affect the mechanical properties of the TMCs due to the exothermic reaction of the TiC synthesis caused by the intense heat that is released during the deposition of TMCs by direct energy deposition using a high-power laser source. Thus, the increased absolute temperature weakens the surface tension due to gradients in interfacial tension. This occurrence may promote the expansion of the molten pool and may increase the Marangoni effect velocity. In addition, in cases of a greater amount of TiC, due to the thermal instability, the primary dendritic phase becomes coarser and influences the overall properties of TMCs. Therefore, the agglomeration of the solute element is reduced, resulting in a dispersed distribution of the reinforcements, as can be seen in Figure 9 and Figure 10. Additionally, the TiC-rich samples present a large number of unmelted particles, which are considerably larger in size. This reinforcement phase acted as nucleation sites and refined the matrix microstructure, with the disadvantage of possible micro-crack center propagation. However, when the TiC content was increased over wt.% 3, pores and micro-pitting effects were observed in the volume of the unmelted particles.

### 3.3. Microhardness Assessment

**Figure 11:**
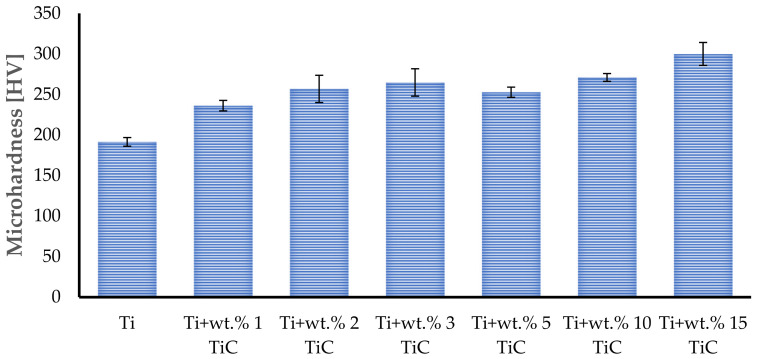
The effect of TiC particle content on microhardness of the deposited sample by DED.

The microhardness values of the samples obtained under optimal processing conditions are shown in Figure 11, and it can be seen that they increased from 192 ± 5.3 HV_0.2_ (pure Ti) up to 300 ± 14.2 HV_0.2_ (Ti + wt.%15 TiC). The microhardness increases with the addition of TiC particles due to the macro-homogeneous incorporation of in-situ synthesized TiC reinforcement throughout the Ti matrix, which induces a strengthening effect. The improvement in hardness observed with the increase in TiC concentration is attributed to the dislocation fixation effect of the TiC particles [43]. At the same time, the fine grain microstructure, due to the fast cycles of heating and cooling specific to the DED process, can increase the hardness of materials, according to the Hall-Petch theory [44,45]. The hardness of the undissolved TiC particles in the Ti matrix fluctuates between 3107.2 ± 88.12 HV and 3254 ± 20.09 HV. Due to the multilayer scanning strategy, the DED-deposited structure is subjected to repeated heating and cooling cycles, which leads to increased hardness due to the smaller grain’s microstructure. Also, the improved values of microhardness in the case of TMCs as compared to pure Ti are provided due to the solid solution strengthening of carbon and the fine grain strengthening. These TiC microstructures can withstand loads orders of magnitude higher than the metal matrix and thus contribute to the increase in resistance to plastic deformations during microindentation tests.

The microhardness of the hypoeutectic TMCs region, according to the Ti-C phase diagram, was measured to be up to 265 ± 16.88 HV (Ti + wt.% 3 TiC), which was about 35 HV lower than that of the hypereutectic TMCs region, which shows to be 300 ± 14.2 HV_0.2_ (Ti + wt.% 15 TiC). In TMCs with a TiC content up to wt.% 3, the microhardness indenter caused deformation and mobilization of TiC phases, and smaller microhardness was obtained. On the other hand, when the microhardness indenter was applied on the surface of the wt.% 15 TiC TMCs, the larger primary TiC dendrites acted as a reinforcement to the material deformation and were less deformable.

In comparison to titanium fabricated via powder metallurgy, this paper demonstrates a significant increase in microhardness, with a maximum value of around 300 HV compared to approximately 176 HV [46]. Studies have shown that commercially pure titanium without any surface treatments typically has a microhardness of ~200 HV [46,47,48]. Researchers have reported that under different experimental conditions and with varied powder morphologies, titanium metal matrix composites (TMCs) exhibited a microhardness of 312 HV at approximately 20% TiC volume fraction [49]. Also, another DED sample with a 20% TiC deposition showed an increase in hardness at 350 HV [12]. Thus, the TMCs studied in this paper offer notable advantages in terms of microhardness compared to similar composites in the literature.

### 3.4. Friction and Wear Behavior of TMCs

**Figure 12:**
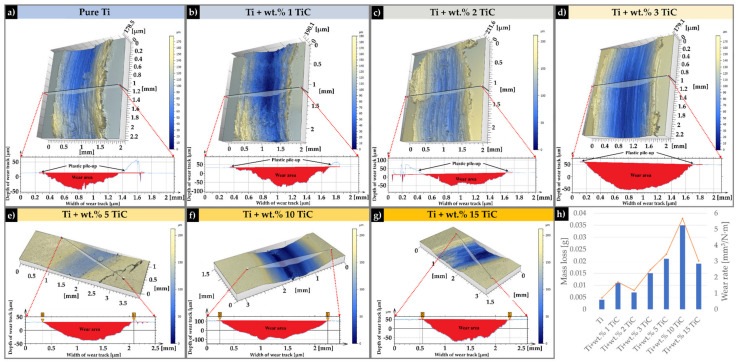
The 3D surface profiles of the Ti and TMCs samples fabricated by DED after wear testing against their steel ball counterparts, the matching 2D profile of the wear track and the corresponding mass loss and wear rate of each sample and the graphical representation of the results (**h**).

The wear track morphologies of TMCs with different TiC content were analyzed using the non-contact optical profilometry technique, as presented in Figure 12. The wear track width and depth of the hypoeutectic TMCs region were considerably greater than those of the hypereutectic area. To provide a certain observation of the differences of the wear scar, cross-section profiles were measured across wear tracks and are presented in Figure 12. For the samples with a TiC content of up to wt.% 3, the wear tracks reveal a maximum width and depth of 1.53 mm and 107 μm, respectively (Figure 12d). The circular wear reciprocating test showed significant tribological differences between the TMC samples with different content of reinforcement TiC particles and the pure Ti sample tested at room temperature. Figure 12a reveals a low value of the Ti-deposited sample wear scar with a depth and width of 57 µm and 1.07 mm, respectively. The geometric characteristics of the wear scar in the case of the Ti + wt.% 1 TiC sample (Figure 12b) were two times deeper, reaching up to 112 µm depth and 1.29 mm width. When increasing the TiC content to wt.% 2, the wear scar depth was 72 µm and width 1.15 mm (Figure 12c). In addition, plastic deformation pile-ups were observed at the edge of the wear tracks of samples with wt.% 1, 2, 3 TiC content, which indicates excellent plasticity.

In the case of wt.% 5 TiC, the wear track was more accentuated with deeper groves that reach up to 139 µm depth and 1.721 mm width (Figure 12e). The increase in carbon due to the step-up to wt.% 10 TiC provides the necessary mass for increasing the depth and width values up to 206 µm and 1.98 mm (Figure 12f). This phenomenon was not further magnified in the bulk sample with wt.% 15, that reach to a depth of 121 µm and a width of 1.68 mm. In contrast, with the sample with wt.% TiC up to 3%, no plastic deformation pile-up was observed.

The wear rate is not directly proportional with the rise content of TiC reinforcement [50]. The depth of the tracks does not evolve in a linear manner and may be attributed to the complex processes occurring during the wear of the composites.

Based on the wear depth and the mass loss, the wear rate can be calculated by Equation (2):

(2) $$W_{r}=\frac{V}{F\cdot S}$$

where W_r_ is the wear rate (mm^3^/N·m), V is the wear volume (mm^3^), S is the total friction distance (m), and F is the load (N). The wear rate graphs of each sample are presented in Figure 12h and show how it changes with the addition of TiC dispersed phase.

The analyzed results showed that the average wear volume of the sample fabricated of pure titanium was about 0.71 mm^3^/N·m, while the average specific wear rate for the composite sample mixed with wt.% 1, wt.% 2, and wt.% 3 TiC was about 1.7 mm^3^/N·m, 1.18 mm^3^/N·m, and 2.45 mm^3^/N·m, respectively. In the case of TMCs with wt.% 5 TiC, the wear rate was 3.43 mm^3^/N·m. When the content of TiC was increased up to wt.% 10, the wear rate also increased to 5.67 mm^3^/N·m. The increase in Carbon due to the step-up to wt.% 15 TiC provides the necessary mass to decrease the wear rate to 3.02 mm^3^/N·m.

The worn surfaces of Ti, Ti + wt.% 1, Ti + wt.% 2, and Ti + wt.% 3 TiC are presented in Figure 12a–d. A large number of ridges and micro-plow structures are observed on the worn surfaces of bulk-deposited materials under a load of 60 N, which significantly contributed to the increased surface roughness and frictional resistance. The worn surface of each analyzed sample exhibits complex and severe wear tracks. As can be seen from Figure 12, a large number of plowing grooves and spalling pits by stick-slip worn mechanism were observed on the analyzed surface. Edges produced by abrasive impurities that were caught between the sample surface and steel ball during the friction investigations together with microplow structures resulting from considerable plastic deformation during the wear examination were also observed. The presence of wear grooves and debris indicates the apparition of abrasive wear during the testing.

The microstructure of the TMCs with a TiC content up to wt.% 3 presented small pits that can be observed on the wear surface, which were caused by the granular eutectic, primary, and chain-shaped eutectic TiC microstructures that were being pulled out during the wear test. These pulled-out TiC particles became part of the wear debris, contributing to a reduction in the value of friction coefficients COF). These pulled-out TiC contents that caused large-scale spalling and plastic deformation would make the debris mix into the friction pair, contributing to a transition in the wear mechanism of TMCs from two into three friction elements with additional debris. The combination of these factors decreases the wear resistance.

The wear marks of the hypereutectic TMCs region (wt.% 5, 10, and 15 TiC) presented no pits, thus indicating that the large dendritic TiC phases were not contributing to the surface wear. This can be attributed to the larger size and deeper embedment of the primary dendritic TiC within the Ti matrix compared to those phases of TiC that are presented at a low amount of C. However, the remaining dendritic TiC caused a rough wear surface, leading to an increase in the coefficient of friction.

The wear performance of TMCs was also investigated by Zheng Y. et al. [51]. He discovered that the morphologies of ceramic particles significantly influence the wear resistance. TMCs with equiaxed TiC presented a higher wear rate, and TMCs with dendritic TiC led to an improvement in wear resistance due to the two-body wear mechanism instead of the one with three bodies.

**Figure 13:**
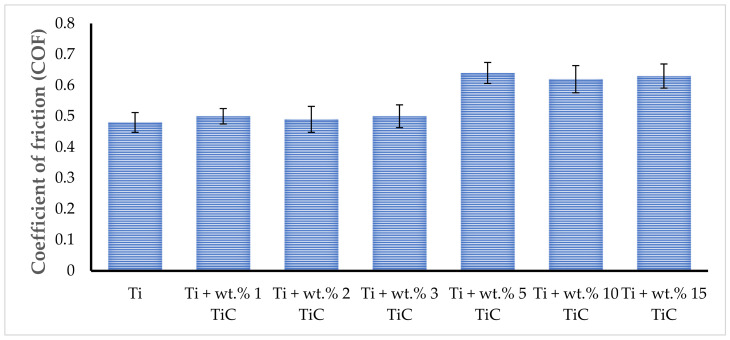
The mean value for coefficient of friction for each deposited sample.

The control sample (titanium) displayed a high and unstable friction coefficient when sliding against the steel ball. The friction trace of TMCs rotating against steel balls in the air can slightly fluctuate during the complete testing period because of the stick-slip adhesive wear. The physical interaction between the testing surface and counterbody surface increases when increasing tangential force over junction growth, and both contact surfaces remain in adhesion, showing a ‘stick’. Then, as the applied force exceeds the adhesive strength, the junction ruptures and a rapid ‘slip’ for both surfaces can occur. This stick-slip series happened repetitively, accounting for the friction force fluctuation. The mean values for the friction coefficient of TMCs with different TiC morphologies were calculated, and the results are presented in Figure 13. The COF average was between 0.48 ± 0.032 (pure Ti) and 0.63 ± 0.039 (Ti + wt.% 15 TiC). A high COF is generally associated with a large wear rate due to the high adhesion rate on the counterpart. The TMCs that are in the hypoeutectic region, despite having a lower COF, exhibited a wear rate of 2.45 mm^3^/N·m (Ti + wt.% 3 TiC) compared to the ones in the hypereutectic region, which measured a COF of 3.02 mm^3^/N·m (Ti + wt.% 15 TiC). Therefore, the friction and wear performance cannot be exclusively evaluated based on the value of the COF.

### 3.5. Tensile Properties

**Figure 14:**
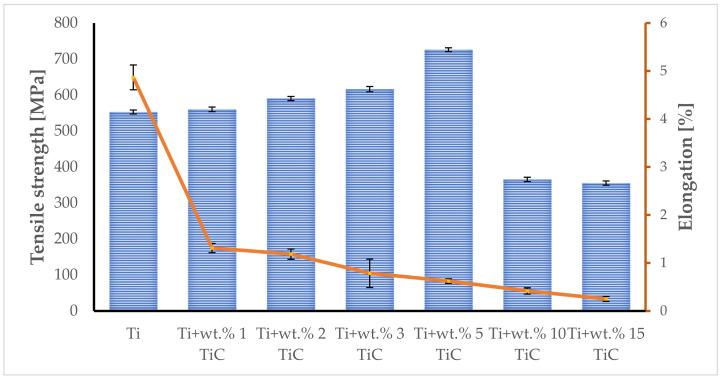
Comparison between the tensile strength and elongation of each TMC composition-deposited sample.

Commercially pure Ti showed a significant plastic deformation, but after adding TiC to the composition, the fracture feature of TMCs drastically changed, becoming brittle, as presented in Figure 14. The average ultimate tensile strength and elongation of pure Ti fabricated by DED are approximately 552.8 ± 5.5 MPa and 4.87 ± 0.25%, respectively. The tensile properties, which consist of tensile strength and elongation of TMC with wt.% 1 TiC, are approximately 560 ± 6.2 MPa and 1.31 ± 0.09%, respectively. When the TiC content was increased up to wt.% 2, the tensile strength improved to approximately 590 ± 5.8 MPa and elongation decreased to 1.18 ± 0.1%, which translates into more brittle structures. The TMC with wt.% 3 TiC followed the same trend as before, where the tensile strength increased to 616 ± 7.4 MPa and elongation was diminished to 0.79 ± 0.29%. The results obtained in case of the TMC with wt.% 5 TiC maintain the increasing tendency in case of tensile strength by rising to 725 ± 5.4 MPa and become even more fragile compared with the samples with TiC content between wt.% 1–3, which had a maximum elongation of 0.62 ± 0.04%. The raised content of Carbon up to wt.% 10 TiC provides the necessary mass for changing the TiC reinforcement microstructure to primary TiC, leading to a reduction in tensile strength and elongation to 365 ± 6.1 MPa, and 0.42 ± 0.06%, respectively. When the content of TiC increases up to wt.% 15 TiC, this phenomenon intensifies, exhibiting a diminution of tensile strength and elongation down to 355 ± 5.9 MPa and 0.25 ± 0.05%. In conclusion, an amount of wt.% 5 TiC can improve tensile strength, but with the limitation that the plasticity of the Ti matrix is drastically reduced.

Experimentally, it was demonstrated that the melting mechanisms in the case of TiC particles after laser irradiation are (i) epitaxial growth along the margin of the partially melted TiC particle and (ii) complete dissolution of TiC particles. However, when we refer to the thermal behavior of TiC, the maximum temperature was located in the core of the particles, which was diminished gradually when reaching the outer shell surface. The transformation of the TiC phases is directly influenced by the C percentage. In case of a complete melting, a transformation to a primary dendritic phase may occur, and the coarse type forms secondary and tertiary dendrites along its margins due to the instability of the thermal gradients.

**Figure 15:**
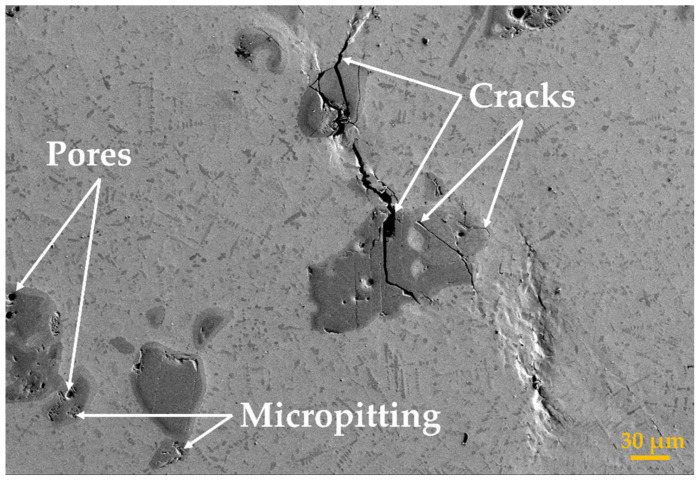
Microcrack propagation and defects in form of pores and micropitting.

The TiC particles dispersed in the TMC matrix strengthen the fracture mechanism as part of the force from the base matrix that is retrieved by the reinforcement TiC phase. The principal feature that leads to a reduced tensile strength factor of the deposited samples when the TiC content was above wt.% 10 was caused by the apparition of premature cracking. As is known, when components are under load, the brittle materials can only relieve external stresses by cracking, whereas in ductile materials the stress relief can be accomplished by plastic deformation. On the other hand, by increasing the TiC content, there is less ductile matrix between brittle phases; thus, more micro-cracks are susceptible to propagation, which finally leads to reduced material strength. If the bonding between the TiC particles and Ti matrix is strong enough, after applying an external stress to the sample, the load could be transferred from the matrix to the TiC phases. Thus, the brittle cracks would form inside the TiC phases or in the vicinity of unmelted particles (Figure 15). In the case of using TiC particles, the obtained TMC samples present a larger interfacial area between the TiC and Ti matrix and smaller inter-particle spacing for a given particle volume fraction, which is advantageous for obtaining more load transfer from the matrix to the TiC reinforcements, thus leading to an increased value in tensile strength. However, when the TiC content goes beyond wt.% 10, the size of dendritic primary TiC becomes substantially larger, and some unmelted particles exhibit pores or a micropitting effect. More fractured, unmelted particles and dendritic primary TiC phases remain on the fracture surfaces as the TiC content increases, leading to a less resistant structure. During the stretching process, the unmelted particles and dendritic primary TiC phases with larger size hinder the plastic deformation of the Ti matrix, causing stress to be concentrated in their vicinity. In addition, microcracks are prone to generate, extend, and merge in their vicinity, reducing the strength and ductility of the TMCs and making them susceptible to premature cracking. Thus, this indicates that when a high proportion of TiC is added (beyond wt.% 10), the bonding strength between TiC and the Ti matrix is still achievable and TMC will fail in terms of tensile strength. Moreover, since the ductility of the matrix decreases, the crack spreads more easily. The rupture mechanism of this type of material was also investigated by Yu C. et al. [30], but at a content of wt.% 20 TiC and processed in different experimental conditions. He also concluded that only by reducing the content and size of unmelted TiC particles and the dendritic primary phase can the tensile properties of TMC be improved.

The lack of cohesion of the interface between TiC particles and the Ti matrix influences the tensile properties of the TMCs. The bonding strength of the interface between reinforcement particles and matrix is influenced by the diameter of the strengthening particles. If the dimensions of these particles increase, the possibility of fully melting them is reduced, and the bonding process can be incomplete due to the lack of adequate adhesion at the interface. The behavior of composite materials is often sensitive to changes in temperature. First, the response of the titanium matrix to an applied load is temperature-dependent, and, on the other hand, the temperature changes can cause internal stresses, established because of differential thermal contraction and expansion of the involved elements.

### 3.6. Influence of TiC Percentage on the Thermo-Cycle

**Figure 16:**
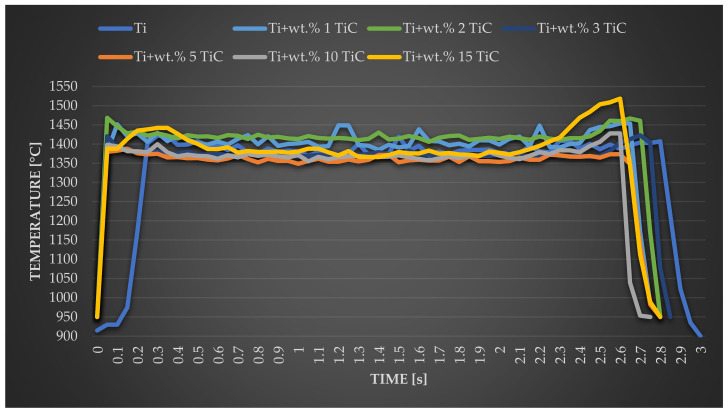
Thermo-cycles of 20 mm length deposited tracks.

Figure 16 displays a typical thermo-cycle of the molten pool, recorded using the IR thermal camera, during the 20-mm-long deposition of commercially pure Ti and the six TMCs with different TiC percentages (wt.% 1, wt.% 2, wt.% 3, wt.% 5, wt.% 10, and wt.% 15), using the same optimized process parameters. This analysis was suitable for revealing the temperature evolution during the deposition process.

At the beginning of the process, the temperature slightly fluctuates from 1400 °C to 1460 °C, probably due to a small stationary period of the robotic arm after the laser starts. After this point, the temperature decreases and remains approximately constant during the entire deposition process, showing only a slowly increasing increase at the end of the deposition, again most probably caused by the reduced speed of the robotic arm. From the plot (Figure 16), it can be observed that in the case of the addition of wt.% 1 and wt.% 2 of TiC particles, the temperature increases up to 1423 °C, while for wt.% 3 of TiC, the temperature tends to decrease at 1377 °C, which is a similar interval compared to the pure Ti sample. During the deposition of TMCs, the small fluctuations of the temperature values can be explained by the fact that a part of the laser beam is absorbed by the TiC particles, which results in a higher melting point (~3140 °C) compared to the pure titanium melting temperature (~1668 °C) and leads to a destabilized melt pool. Overall, during the processing, it can be observed that the absorption of the laser beam was relatively constant, resulting in a steady thermal distribution that ultimately generates a stable melt pool that leads to a structure without defects and a homogeneous distribution of TiC in the titanium matrix.

From the plot interpretation, it can be observed that an increased amount of TiC in the TMCs composition causes a higher molten pool temperature when the TiC reaches up to wt.% 2. This is caused by the presence of TiC particles, which act as heterogeneous nucleation spots. In addition, at this rate of TiC content, the laser beam had sufficient energy to melt the majority of the particles, thus leading to an increase in the temperature values of the melt pool.

The plot of the temperature’s evolution recorded during the LMD process for 20 mm-long deposited tracks of Ti and TMCs with wt.% 5, 10, and 15 TiC concentrations, using the mentioned optimal parameters described in this study, is also displayed in Figure 16. When pure titanium was deposited, temperatures quickly reached a range of 900 °C to 1400 °C within 0.2 s, exhibiting fluctuations between 1370 °C and 1420 °C throughout the deposition process. After the cease of the laser action, the temperature decreased from 1410 °C to below 900 °C in approximately 0.2 s. Extending these observations to the behavior of TMCs, a rapid temperature surge from 900 °C to 1390 °C within less than 0.05 s at the onset of the process was followed by expedited cooling subsequent to the ending of laser interaction. It is noteworthy that the TiC content within TMC of varying concentrations did not have a substantial influence on the temperature evolution during the laser metal deposition (LMD) process. Notwithstanding, an elevation in TiC concentration resulted in a shift in the temperature range. Specifically, with a TiC concentration of wt.% 5, temperature oscillations were noted within the 1350 °C to 1390 °C range, while a wt.% 10 TiC composition yielded fluctuations between 1350 °C and 1430 °C, while for a wt.% 15 TiC composition the measured temperature displayed values within the 1350 °C to 1520 °C range. Consequently, although the minimum temperature remained unaltered, the maximum temperature increased by 40 °C for TMC with wt.% 10 TiC in comparison to that with wt.% 5 TiC and by over 90 °C for TMC with wt.% 15 TiC compared to the sample with wt.% 10 TiC. Therefore, we may conclude that the TiC particles increase the amount of stored heat and slightly decrease the heat dissipation rate of the liquid phase because it takes a longer time for the cooling to take place after the laser beam is turned off.

The decrease in maximum temperature of the melt pool was also influenced by the fact that the thermal conductivity of Ti is lower than the one of TiC. The average temperatures of the deposited layers with different TiC percentages indicate a good metallurgical bond between the deposited layers and the TiC microstructure with the Ti metal matrix. The thermal signature analysis can offer a better understanding of the correlation between molten pool characteristics with input processing parameters and can be used to predict the microstructural evolution of TiC.

## 4. Case Study

**Figure 17:**
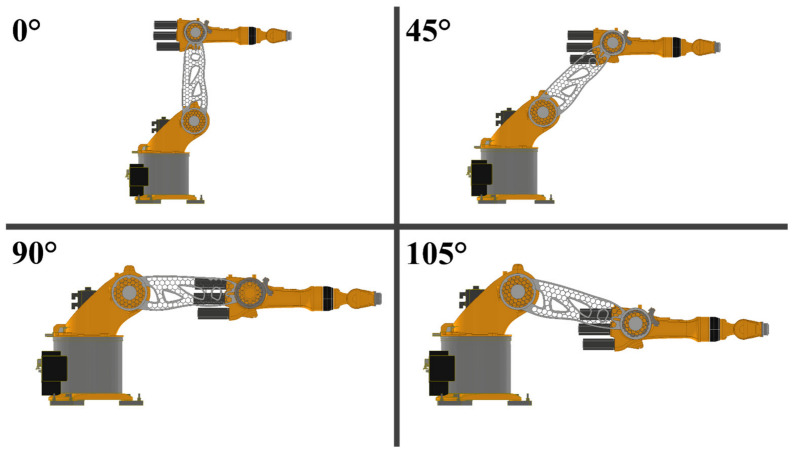
3D modeling of a median robotic arm at different working positions.

To demonstrate the industrial use of the method, a 3D model of a robotic arm for industrial application was modeled in a computer-aided design (CAD) program (Figure 17) and was subsequently manufactured by 3D additive manufactured using the DED method from a TMC using commercially pure Ti as matrix with wt.% 5 TiC reinforcement particles. In general, this type of robot uses a side-mounted or a pendulum assembly so that the operating range of the arms is large enough. Under this circumstance, the operating range can achieve an integrated circle suitable for some complex processing operations. Among all robot components, the middle arm, which is the main moving component, occupies a large weight and volume. Its weight has a significant effect on the movement precision, processing time, costs, and product life cycle. Therefore, to overcome these limitations, TMCs can be used to increase the mechanical properties of the arm by increasing its hardness, tensile strength, and wear resistance.

**Figure 18:**
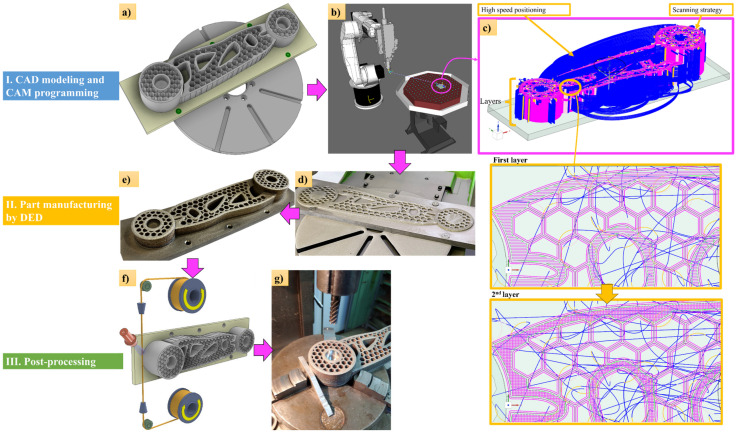
The technological flow of obtaining the pilon bracket via LMD: CAD model (**a**), CAM program (**b**), scanning strategy (**c**), part fabrication (**d**,**e**) and post-processing stage (**f**,**g**).

The technological flow for the manufacturing of the robotic arm in the industrial field is presented in Figure 18. and consists of the following stages: First, the CAD model was designed (Figure 18a), then it was imported into a computer-aided manufacturing (CAM) program (Figure 18b), and the best scanning strategy was selected from the CAM program for the specific layer-by-layer manner of DED printing (Figure 18c). After the positioning and fixing step of the substrate, along with the testing of the executed trajectories on the DED machine to ensure compliance with the dimensional constraints of the substrate, the actual manufacturing process began (Figure 18d). At the end of the robotic arm printing process, the part was removed from the substrate by wire cutting (Figure 18f) and milled the functional surfaces (Figure 18g).

## 5. Conclusions

Different types of TMC samples were manufactured successfully by direct energy deposition technique using a high-power laser with different compositions (wt.% 1, wt.% 2, wt.% 3, wt.% 5, wt.% 10, and wt.% 15 TiC). The laser method of manufacturing TMCs promotes the dissolution of TiC particles and thus strengthens the composites. The powder material dimensions and processing parameters employed in this study have contributed to the development of the morphology of the reinforcing phase, which varies with TiC content. This distinct morphology differs from those documented in existing literature that uses the same amount of TiC. The main conclusions are as follows:

- The phase compositions of different TMCs (TiC/Ti) consist of TiC, α-Ti, and β-Ti. The morphology of TiC in the hypoeutectic region (with a TiC content under wt.% 5) is as follows: granular and chain-shaped eutectic TiC, eutectic TiC, granular primary TiC, and a reduced number of unmelted particles. In the case of TMCs that are in the hypereutectic region (TiC content over to wt.% 5), the possible TiC morphologies are: granular primary TiC, granular and chain-shaped eutectic TiC, primary dendritic TiC, and coarse primary dendritic TiC with secondary and tertiary dendrites due to the thermal instability of the melt pool. The chain-shaped eutectic TiC morphology has the advantage of reducing the rapid propagation of cracks in the TMC structure.
- The strengthening effects such as fine grain strengthening and solid solution strengthening have a significant impact on the Ti matrix, increasing the strength of TMCs up to 725 ± 5.4 MPa but drastically decreasing elongation to 0.62 ± 0.04%. Thus, adding a small amount of TiC, up to wt.% 5, can improve tensile strength, but with the limitation that the plasticity of the Ti matrix is considerably reduced.
- Increasing the TiC amount in the matrix raises the TMCs hardness, with values of up to 300 ± 4.3 HV_0.2_ in the case of wt.%15 TiC vs. 192 ± 5.3 HV_0.2_ in the case of pure Ti. All samples with TiC content exhibited higher values of hardness as compared to the pure Ti matrix manufactured with the same parameters.
- The wear rate is not proportionally affected by the content of TiC reinforcement. The depth of the tracks does not evolve in a linear manner and may be attributed to the complex processes occurring during the wear of the composites. The TMCs that are in the hypoeutectic region exhibit a wear rate of 2.45 mm^3^/N·m (Ti + wt.% 3 TiC) and an average friction coefficient (COF) of 0.48 compared to the ones in the hypereutectic region, which measures a wear rate of 3.02 mm^3^/N·m (Ti + wt.% 15 TiC) and a COF of 0.63.

Overall, the material with wt.% 5 TiC content in the Ti matrix showed superior mechanical properties in comparison with the pure Ti: uniform distribution of elements, higher hardness, and greater tensile strength.

In terms of future plans, based on the findings of our study regarding the correlation between particle shape and TiC phase morphologies, particularly in relation to the TiC volume percentage, the authors plan to develop a numerical simulation code. This will facilitate the precise prediction of the microstructure of TMCs, with a focus on the pursued mechanical aspects.

## Figures and Tables

**Table 1:** Physico-thermal properties of Ti and TiC.

Material	Density [kg/m^3^]	Melting Temperature [°C]	Thermal Conductivity [W/m·K]	Hardness [HV]	Crystalline Structure
Ti	4500	1668	17	117–202	α-HCP; β-BCC
TiC	4940	3160	21	2500	FCC

**Table 2:** Mechanical—physical properties of Titanium and Titanium carbide.

Material	Density [g/cm^2^]	Melting Point [°C]	Hardness [HV]	Young Modulus [GPa]	$\mathrm{Coefficient}\;\mathrm{of}\;\mathrm{Linear}\;\mathrm{Thermal}\;\mathrm{Expansion}\;[\times 10^{-6}\;{}^{\circ}C]$	Thermal Conductivity [W/m·K]
Ti (99.9% purity)	4.51	1668	134	120	8.6	11.4
TiC	4.93	3140	3200	460	7.61	17.16

## Data Availability

The original contributions presented in the study are included in the article, further inquiries can be directed to the corresponding authors.
